# Use of Multi-Hollow Polyester Particles as Opacifying Agent for Injection-Molded Polyethylene

**DOI:** 10.3390/polym12061331

**Published:** 2020-06-11

**Authors:** João F. A. Silva, Tânia M. T. Carvalho, Margarida L. Almeida, Paula Pereira, Helena Sousa, Jorge Martins, Luísa Carvalho, Fernão D. Magalhães

**Affiliations:** 1 LEPABE—Laboratory for Process Engineering, Environment, Biotechnology and Energy, Faculty of Engineering, University of Porto, Rua Dr. Roberto Frias, 4200-465 Porto, Portugal; jmfas@fe.up.pt (J.F.A.S.); taniamtcarvalho@fe.up.pt (T.M.T.C.); lhcarvalho@estv.ipv.pt (L.C.); 2 DEMAD, Department of Wood Engineering, Polytechnic Institute of Viseu, Campus Politécnico de Repeses, 3504-510 Viseu, Portugal; malmeida@fe.up.pt (M.L.A.); jmmartins@estgv.ipv.pt (J.M.); 3 Isolago, Estrada Nacional 3–km 16, 2050-038 Aveiras de Baixo, Portugal; paula.pereira@componit.pt (P.P.); helena.sousa@isolago.com (H.S.)

**Keywords:** opacifier, opacity, thermoplastic, hollow particles

## Abstract

Titanium dioxide is considered the most efficient white pigment for opacification of thermoplastics. However, its high cost, combined with strong price oscillations due to production bottlenecks, has been driving the industry towards alternatives that might allow reducing the titanium dioxide content, while maintaining the product’s opacity. A strategy commonly used in waterborne paints consists in adding hollow polymer particles to the formulation, therefore achieving opacification due to light refraction at the air/polymer interface. In the current work, we show preliminary results that indicate that a similar strategy can be followed for thermoplastics opacification, as long as thermoset particles are used, in order to ensure preservation of the hollow geometry during melt-processing. Multi-vesiculated crosslinked styrene–polyester particles, produced by a single-step double emulsion process, are used. Evidence of synergic interaction between the multi-hollow particles and titanium dioxide has been found.

## 1. Introduction

Titanium dioxide (TiO_2_) is the most used white opacifying pigment in the coatings and thermoplastics industry. The effectiveness of titanium dioxide particles in conferring opacity results from its excellent light scattering properties. TiO_2_ refractive index (2.7 for the rutile crystalline form) is relatively large in comparison to the polymer materials used as binders in a coating or as thermoplastic materials (typically 1.5), causing effective light scattering within the polymer. In addition, TiO_2_ particles are available with average particle sizes of 0.2 to 0.3 μm, corresponding to about half the wavelength of yellow-green light, which is the most easily detected by the human eye. That radiation is therefore diffracted by the TiO_2_ particles dispersed in the polymer matrix, complementing the light refraction effect [1,2].

Due to production bottlenecks combined with large demand, the cost of TiO_2_ has been high and undergoing significant oscillations. This has motivated the industry to research alternatives, in order to reduce the amount of TiO_2_ needed for product opacification. Inorganic pigments most used nowadays for partial replacement of titanium oxide are zinc oxide and lithopone (a mixture of barium sulfate and zinc sulfide), with refractive indexes of 2.02 and 1.84, respectively [3]. Total replacement of TiO_2_, however, is not feasible, since, in order to obtain equivalent opacity, it would imply producing materials with about four times larger thickness [4].

However, the opacity of a polymer material can also be increased by occluding air-filled voids, taking advantage of the difference in refractive index between air and polymer. This principle has been successfully used in the coatings industry since the 1980s, in the form of hollow polymer microparticles that can be easily incorporated in a waterborne paint formulation [5,6,7,8]. However, these particles are typically made of polystyrene and acrylic polymers, with glass transition temperatures at around 100 °C. This makes them inappropriate for use in opacification of thermoplastic matrixes that are processed at higher temperatures, since they would break or collapse under shear and lose the geometric integrity of the internal voids.

In this work, we investigate the potential of using multi-hollow particles (MHP) based on styrene-crosslinked unsaturated polyester produced by a one-step double emulsion process. These particles have been studied before by our group as opacifiers for waterborne paints [9,10,11,12], but it is the first time that they are proposed for thermoplastics. Since they are made of a rigid thermoset material, the void geometry was expected to be preserved during melt processing.

## 2. Materials and Methods

### 2.1. Materials

The unsaturated polyester used was provided by Omnova Solutions (Mem Martins, Portugal) as 70 wt.% solution in styrene. According to the provider, it has an acid value of 15.5 mg KOH/g and a Brookfield viscosity of 1570 mPa.s. Poly(vinyl alcohol) (MW = 205000 g·mol^−1^) was also provided by Omnova Solutions (Mem Martins, Portugal). Styrene (4-tert-butylcatechol stabilizer) and 1,3 diaminopropane were supplied by Sigma-Aldrich (St. Louis, Missouri, USA), with certified mole fraction purity of 0.999. Dibenzoyl peroxide 75 and maleic anhydride 99% (pellets) were supplied from Acros Organics (Geel, Belgium). Low-density polyethylene (SABIC LDPE 1695N0) and titanium dioxide (TRONOX CR-470) were supplied by Isolago (Pontével, Portugal).

### 2.2. Multi-Hollow Particles Production

The multi-hollow particle synthesis process adopted here was the single-step double emulsification procedure described in previous works [9,10,11]. Two differences were introduced in relation to the standard formulation previously described, in order to minimize the yellowish tone of the final particles: diethylenetriamine was replaced with 1,3-diaminopropane amine, and the curing system composed of cumene hydroperoxide and ferrous sulfate was replaced with dibenzoyl peroxide. First, the organic phase, a solution of polyester in styrene, was neutralized with 1,3-diaminopropane amine, and then diluted with additional 30 wt.% styrene. 3 wt.%. dibenzoyl peroxide was added to the organic phase as radical initiator for the final curing step. The organic phase was then gradually emulsified into the aqueous phase containing poly(vinyl alcohol) (2.6 wt.%) and 1,3-diaminopropane amine (0.9 wt.%). Mixing was performed in a jacketed glass 1000 mL reactor at constant temperature (20 °C) under mechanical stirring (1100 rpm) for 60 min, using a 55 mm diameter Cowles-type impeller. Finally, the water/oil/water double emulsion droplets were cured in order to yield solid hollow polyester particles. For this, the reactor temperature was raised to 70 °C and mechanical stirring decreased to 300 rpm and maintained for 120 min. The solids content of the final waterborne dispersion was 28 wt.%. The particles were then allowed to deposit for a week, and afterwards removed and dried in an MMM (Munich, Germany) Vacucell vacuum oven for 48 h at 65 °C and 0.2 bar.

### 2.3. LDPE Samples Production

Opacity agents and LDPE were fed into an Xplore MC5 (Sittard, The Netherlands) twin-screw mini extruder set at a temperature of 120 °C and a screw speed of 350 rpm. The materials were mixed for 5 min. Injection molding was performed with a Xplore (Sittard, The Netherlands) IM5.5 to obtain dog-bone shaped samples. The injection unit was set at 120 °C and the mold chamber at 80 °C to obtain dog-bone shaped samples (80 × 11 × 2mm).

### 2.4. Characterization

#### 2.4.1. Particle Size Distribution

To determine the particle size distribution, a LS 230 Particle Size Analyzer (Beckman Coulter, Brea, CA, USA) was used. Produced dispersions of MHP were diluted and placed in a sonicator for 15 min to minimize particle agglomeration, and then, introduced dropwise in the equipment’s cell.

#### 2.4.2. Relative Opacity

The relative opacity was determined for six replicas using a PerkinElmer LAMBDA 750 UV/Vis/NIR spectrophotometer (Waltham, Massachusetts, USA). The measurements were made between 400 and 800 nm for polyethylene specimens with different weight fractions of MHP and/or titanium dioxide. The opacity (*O*) is expressed, according to EN 13292:2007 standard, as:

(1) $$O=\frac{I_{B}}{I_{W}}$$

where *I_B_* is the signal intensity on the black background and where *I_W_* is the signal intensity on the white background. The relative opacity (*RO*) can be expressed as

(2) $$RO=\frac{O_{S}-O_{R}}{1-O_{R}}$$

where *O_S_* is the sample’s opacity and *O_R_* is the reference’s opacity. In this case, the reference is a specimen made with unfilled polyethylene. To obtain the integrated relative opacity (*IRO*) for the whole visible wavelength spectrum, the integral of the curve was weighted by the standard solar spectra given by the ASTM E490 standard, *f(λ)*:

(3) $$IRO=\frac{\sum^{\;}\left(RO\left(\lambda \right)\times f\left(\lambda \right)\right)}{\sum^{\;}f\left(\lambda \right)}$$

#### 2.4.3. Scanning Electron Microscope

Particle morphology and internal vesiculation were observed by scanning electron microscopy (SEM), using a FEI Quanta 400FEG ESEM/EDAX Genesis X4M (Hillsboro, Oregon, USA) at Centro de Materiais da Universidade do Porto (CEMUP).

In order to allow visualization of the particle’s interior, dried particles were encapsulated in epoxy resin, and the resulting composite was fractured in liquid nitrogen after hardening overnight. Before being analyzed, samples were sputtered with gold/palladium using a K575X Sputter Coater by Quorum Technologies (Lewes, United Kingdom).

#### 2.4.4. Differential Analysis Calorimetry

DSC analysis was performed on a DSC 204 F1 Phoenix (NETZCH, Bobingen, Germany). Around 10 mg of sample was scanned from 30 to 150 °C at a heating rate of 20 °C/min and under inert nitrogen atmosphere. The results were obtained from the second heating ramp. The degree of crystallization was computed from the ratio between the measured melting enthalpy and the reference value for holocrystalline LDPE, 293 J/g.

#### 2.4.5. Tensile Tests

The tensile tests of the composite thermoplastic specimens were performed in a Tinius Olsen (Redhill, United Kingdom) H50 KT tensile testing machine with a 10 kN load cell. Dog-bone shaped samples (80 × 11 × 2 mm) were drawn at a speed of 10 mm·min^−1^. The computed results for ultimate tensile strength, percentage elongation at break, and tensile modulus are based on engineering stress and strain.

#### 2.4.6. Accelerated Aging Tests

Laboratory aging was performed using a Q-Lab (Westlake, Ohio, USA) Q-SUN Xe-3 xenon test chamber equipped with xenon arc lamps according to EN ISO 4892-1 and 4892-2 norms. This chamber reproduces the damage caused by full-spectrum sunlight, in this case, filtered by windows glass. The exposure using window glass filters followed the conditions of cycle #5 (method B) of EN ISO 4892-2. The irradiance was (1.1 ± 0.02) W/(m^2^ nm) at 420 nm, the black standard temperature was 65 ± 3 °C, the chamber temperature was 38 ± 3 °C and the relative humidity 50 ± 10%. In order to assess the color difference (Δ*E*) during the aging test, measurements were performed before and after the test, using a Konica Minolta (Tokyo, Japan) CM-2500c spectrophotometer. Δ*E* was then determined according to CIE76 formula:

(4) $$\Delta E_{ab}^{*}=\sqrt{{\left(L_{2}^{*}-L_{1}^{*}\right)}^{2}+{\left(a_{2}^{*}-a_{1}^{*}\right)}^{2}+{\left(b_{2}^{*}-b_{1}^{*}\right)}^{2}}$$

## 3. Results and Discussion

### 3.1. Multi-Hollow Particles

**Figure 1:**
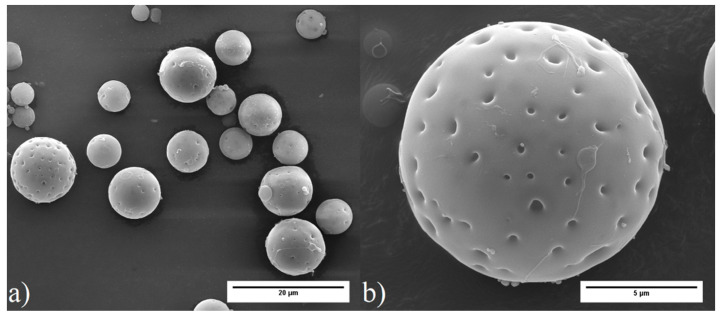
SEM images of MHP (magnification: (**a**) 4000×; (**b**) 16,000×).

**Figure 2:**
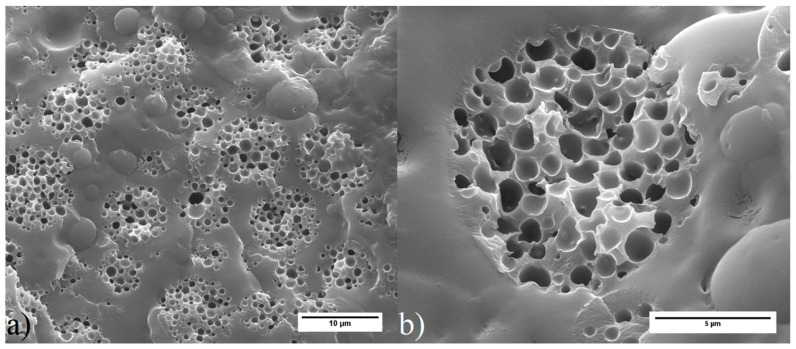
SEM image of MHP embedded in epoxy resin. The sample was fractured under liquid nitrogen, in order to show internal vesiculation of particles (magnification: (**a**) 5000×, (**b**) 15,000×).

Figure 1 shows SEM images of the MHP produced. They are spherical particles with homes on the surface, associated with collapse of the walls of the outer voids. Figure 2a,b show SEM images of MHP embedded in epoxy resin and fractured under liquid nitrogen in order to allow visualization of the particles’ interior morphology. It can be seen that the particles are uniformly vesiculated, with well-defined spherical voids separated by thin polymeric walls.

**Figure 3:**
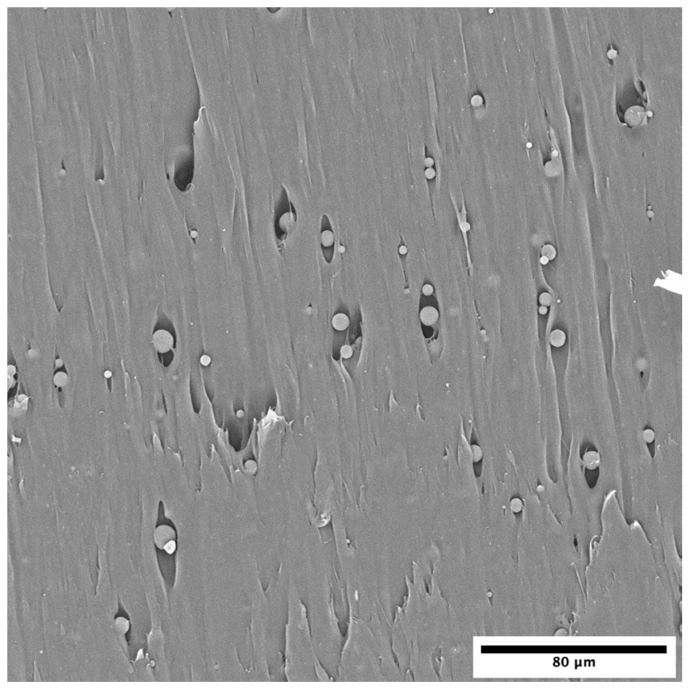
Cross-section of LDPE sample containing 1.5 wt.% MHP, cut with blade, showing that the particles were undamaged by processing and are uniformly distributed within the LDPE sample (magnification: 930×).

Figure 3 shows the fracture surface of a LDPE sample containing 1.5 wt.% MHP. The particles preserved their spherical shape, with no apparent damage being caused by the melt mixture and injection operations. The images also show that the particles are well distributed within the polymer, with no agglomeration.

**Figure 4:**
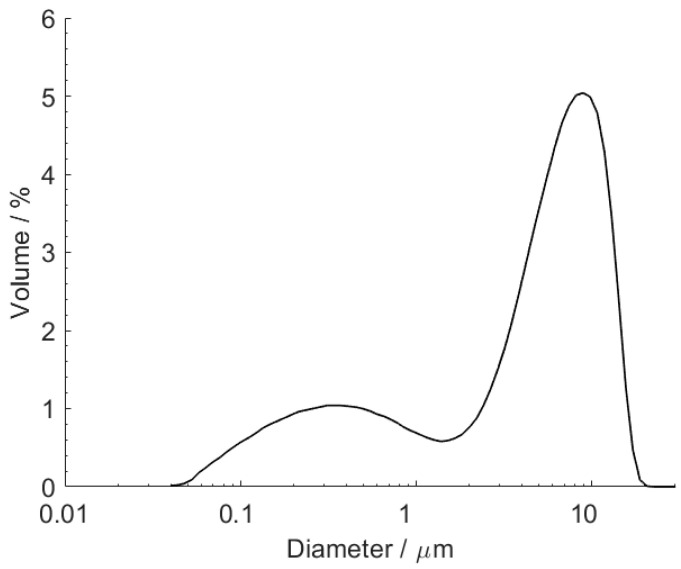
MHP size distribution.

Particle size distribution measurements (Figure 4) show a bimodal distribution, with an average particle size of 5.9 μm. The smaller particles (diameters below 1 μm) are probably not vesiculated, being made of dense polymer.

### 3.2. DSC Analysis

**Figure 5:**
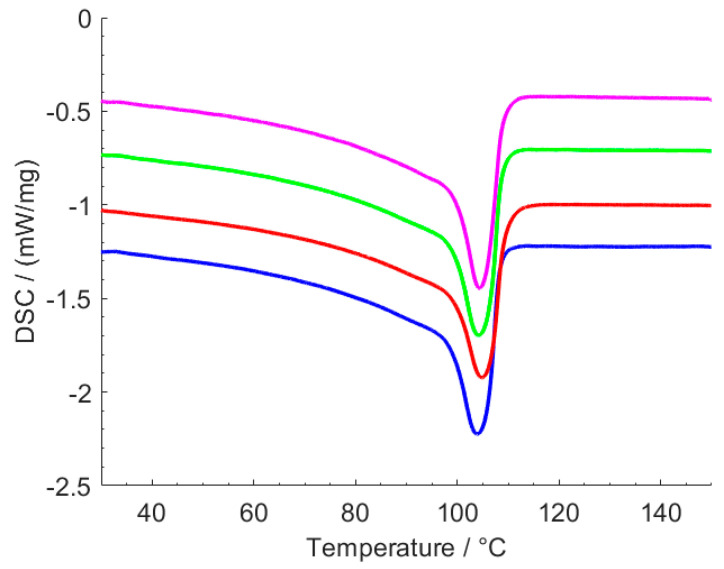
DSC thermograms for (from top to bottom) neat LDPE, LDPE containing 1.5 wt.% TiO_2_, 1.5 wt.% MHP, and 1.5 wt.% TiO_2_ + 1.5 wt.% MHP.

The DSC thermograms obtained for neat LDPE, LDPE containing 1.5 wt.% TiO_2_, 1.5 wt.% MHP, and 1.5 wt.% TiO_2_ + 1.5 wt.% MHP are shown in Figure 5. Table 1 shows the corresponding values of melt peak temperature, melting enthalpy, and percent crystallinity. Both the thermogram features and the percent crystallinity are similar for all samples, showing that the presence of the fillers, for this content level, does not have a significant effect on polymer crystallization. The slightly lower degree of crystallinity obtained for the sample containing 1.5 wt.% MHP is not considered significant. The crystallinity values obtained, around 30%, are considered acceptable, considering that reported values for LDPE typically vary between 30 and 40% [13,14].

### 3.3. LDPE Opacity

**Figure 6:**
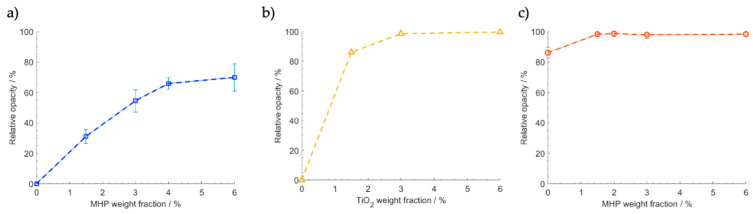
Relative opacity of LDPE as a function of: (**a**) MHP weight fraction, (**b**) TiO_2_ weight fraction, (**c**) MHP weight fraction, with 1.5 wt.% TiO_2_ loading.

To evaluate the performance of MHP as opacifiers and compare it to the one achieved using TiO_2_, LDPE samples with different weight fractions of these materials were prepared. As seen in Figure 6a, samples that only contain MHP achieved a relative opacity of about 70% with 4 wt.% loading, which tends to stabilize at higher MHP content. On the other hand, samples with TiO_2_ alone, shown in Figure 6b, achieve a maximum opacity of 98% with a 3 wt.% loading. This superior efficiency of TiO_2_ was expected. It is well known in the coatings industry that when high opacity is intended, titanium dioxide can only be partially replaced by an opacifier [5,6]. The opacity obtained by combining TiO_2_ at 1.5 wt.% loading with different loadings of MHP is seen in Figure 6c. It is noteworthy that this combination yields an opacity of 98% with 1.5 wt.% TiO_2_ and 1.5 wt.% MHP, while only 86 wt.% opacity was obtained with 1.5 wt.% TiO_2_ alone. Such a difference relative opacity is actually quite perceptible to the naked eye. This signifies that the addition of MHP allows decreasing TiO_2_ content, while still yielding high opacity.

In coatings technology, the improvement of the TiO_2_ opacification effect by addition of a filler is often associated with the latter contributing to improving dispersion and reducing “crowding” of the titanium dioxide particles. The filler particles, called extenders, act as spacers in between the TiO_2_ particles, and thus, improve light scattering efficiency, since individualized particles instead of agglomerated clusters are dispersed within the polymer matrix. However, this effect is only significant when the extender particle sizes are below about 0.5 μm [15]. Relatively large particles like the ones used here should not provide this type of effect. Therefore, we must conclude that the results are due to light refraction caused by MHP’s voids within the polyethylene matrix increasing the incidence of light on the TiO_2_ particles, resulting in a net increase in light scattering efficiency.

**Figure 7:**
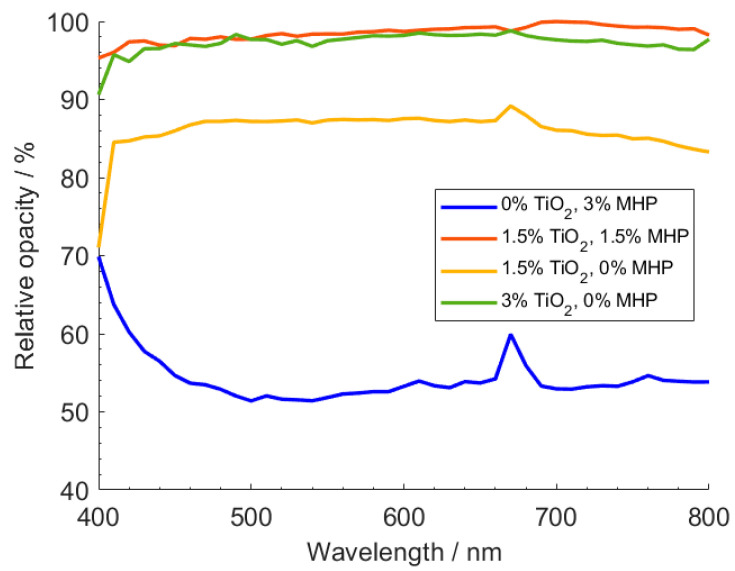
Variation of relative opacity with radiation wavelength for different LDPE compositions.

When observing the relative opacity values as across the visible light spectrum (Figure 7), one can see that the performance of the sample containing 1.5 wt.% TiO_2_ and 1.5 wt.% MHP is indeed similar, across all the spectrum, to the one containing 3 wt.% TiO_2_ alone. On the other hand, the same behavior was not observed in the samples containing only MHP, showing that the opacifying agent has a higher ability to absorb radiation near UV. Thus, when combining both TiO_2_ and MHP, we can obtain a sample that is capable of absorbing radiations homogenously over all the visible spectra.

### 3.4. Mechanical Resistance

To compare the influence of TiO_2_ and MHP on the mechanical properties of polyethylene, tensile tests with samples containing different loadings of both materials were performed.

**Figure 8:**
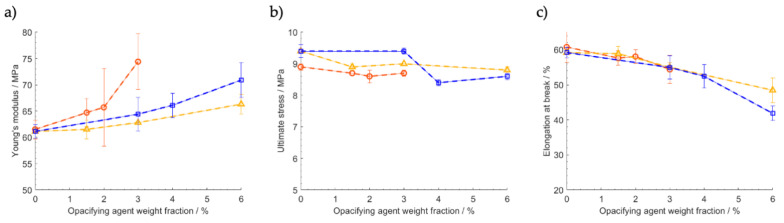
Influence of TiO_2_ and MHP content on mechanical properties of LDPE: (**a**) Young’s modulus, (**b**) ultimate stress, and (**c**) elongation at break. Yellow triangles: samples with varying TiO_2_ content, blue squares: samples with varying MHP content, orange circles: samples with varying MHP content and fixed TiO_2_ content of 1.5 wt.%.

Figure 8 shows that Young’s modulus tends to increase with the increase in loading, showing the expected effect of stiffness increasing with filler content. Concerning tensile strength and elongation at fracture, there is a slight tendency to decrease with loading. However, except for the samples containing MHP at the highest loading, the effect can be considered as hardly significant, considering the observed variability, which is common in this type of measurement. One can conclude that adding MHP to LDPE does not affect significantly its mechanical performance.

The fact that the mechanical performance of the polymer is not affected to a large extent by the incorporation of these fillers, in particular for levels up to 1.5 wt.%, is consistent with the observed calorimetric results. If the degree of crystallinity had changed with filler addition, the mechanical properties would change significantly.

### 3.5. Accelerated Aging

**Figure 9:**
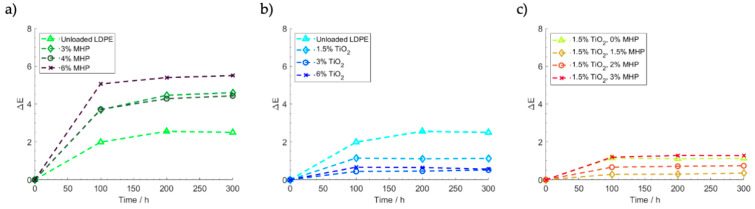
Influence of TiO_2_ and MHP content on color change of LDPE samples during sunlight exposure: (**a**) MHP, (**b**) TiO_2_, and (**c**) MHP and 1.5 wt.% TiO_2_.

Figure 9 shows the color change of the samples when measured after 100, 200 and 300 h simulated sunlight exposure. In all cases, most of the aging occurs on the first 100 h of exposure, and after that, the color tends to stabilize. TiO_2_ has a protective effect on polyethylene due to its UV absorbing properties (Figure 9b), contributing to reduce the color change, while MHP have the opposite effect (Figure 9a), certainly due to their own sensitivity to sunlight. Interestingly, however, when both opacifiers are combined (Figure 9c), MHP intensifies the protective effect of TiO_2_ when both are present at 1.5 wt.% loading. This is once again a consequence of the synergistic effect of MHP increasing the incidence of light on the TiO_2_, increasing its efficiency in absorbing UV radiation. However, for higher MHP contents, the color change increases because of the detrimental effect of the increased MHP concentration.

## 4. Conclusions

This preliminary work demonstrates that an approach based on melt mixing multi-hollow polyester particles with LDPE allows decreasing of the amount of titanium dioxide needed for opacification of the final product. High opacity (98%) can be achieved with only 1.5 wt.% TiO_2_, compared to 3 wt.% when TiO_2_ is used alone. The multi-hollow particles promote light scattering within the polymer matrix, due to the refractive index difference between air and polymer, increasing the incidence of light on TiO_2_, and therefore, resulting in a synergic effect. This approach, based on the occlusion of polymer-encapsulated air voids in a thermoplastic matrix, emulates the long-time use of hollow polymer particles as opacifying agents for waterborne coatings. The fact that crosslinked polyester particles are used, instead of the thermoplastic styrene–acrylic hollow particles traditionally used as opacifying agents for coatings, allows for preservation of the hollow morphology during melt-processing.

## Figures and Tables

**Table 1:** DSC results for LDPE and LDPE with TiO_2_ and MHP fillers.

TiO_2_ Content (wt.%)	MHP Content (wt.%)	Melting Peak Temperature (°C)	Melting Enthalpy (J/g)	% Crystallinity
0	0	104.4	89.6	30.6
0	1.5	104.8	85.5	29.2
1.5	0	104.2	90.3	30.8
1.5	1.5	103.8	90.6	30.9
